# Endometrial Cancer in a Patient With Didelphys Uterus and a History of Renal Cancer: A Case Report and Literature Review

**DOI:** 10.7759/cureus.47114

**Published:** 2023-10-16

**Authors:** Dimitrios Bairaktaris, Eirini Chorianopoulou, Kalliopi Kokkali, Gabriela Stanc, Christos Iavazzo

**Affiliations:** 1 Gynecological Oncology, Metaxa Memorial Cancer Hospital, Piraeus, GRC; 2 Obstetrics, Helena Venizelou General Hospital, Athens, GRC; 3 Pathology, Metaxa Memorial Cancer Hospital, Piraeus, GRC

**Keywords:** diagnostic hysteroscopy, type 1 endometrial carcinoma, endometrial endometrioid adenocarcinoma, mdas, ovarian tumor of borderline malignancy, renal cancer, endometrial cancer, double uterus, didelphys uterus, müllerian duct anomalies

## Abstract

Müllerian duct anomalies (MDAs) concurrent with endometrial cancer are exceptionally rare, with only a few documented cases. Here, we present a case of endometrial cancer in both horns of a didelphys uterus in a 54-year-old woman with a history of renal cancer, who underwent left radical nephrectomy and left salpingo-oophorectomy. The patient sought medical evaluation due to postmenopausal vaginal bleeding. Hysteroscopy with dilation and curettage revealed the presence of two cervixes and two endometrial cavities, with pathology results indicating endometrioid adenocarcinoma (G1). Preoperative MRI staging confirmed the diagnosis of a double cervix and uterus. Subsequently, an open abdominal hysterectomy and a right salpingo-oophorectomy were performed, revealing a didelphys uterus (International Federation of Gynaecology and Obstetrics 2018, stage IA). This manuscript aims to explore the potential correlation between renal and endometrial malignancies in the presence of MDAs.

## Introduction

Müllerian duct anomalies (MDAs) represent rare embryonic malformations of the female reproductive system, stemming from abnormal formation or fusion of the paramesonephric ducts. These anomalies arise between the sixth and twenty-second weeks of gestation, leading to issues such as fusion failure, reabsorption difficulties, or developmental abnormalities of these ducts. The prevalence of MDAs in the general population has been reported to range from 4% to 7% [1]. Among MDAs, the didelphys uterus is classified as a Class III anomaly and accounts for approximately 8.3% of all MDAs, with an estimated incidence of about 3 per 10,000 women [1]. This condition results from incomplete fusion of the Müllerian ducts, giving rise to two separate uterine cavities, each with its own cervical canal. Often, individuals with a didelphys uterus also exhibit two vaginal cavities separated by a longitudinal diaphragm [1]. Grimbizis et al. reported a prevalence of didelphys uterus in fertile women at 4.3%, which closely aligns with the incidence observed in infertile women (3.4%), suggesting that the presence of a didelphys uterus does not significantly impact fertility [1]. Notably, the development of paramesonephric and mesonephric ducts is closely intertwined, leading to urinary tract deformities, such as ipsilateral renal agenesis and duplex collecting systems, in nearly 30% of individuals with a didelphys uterus [1]. However, the association between MDAs and uterine or renal cancer remains relatively unexplored in the literature. To date, only 32 cases of uterus didelphys complicated by endometrial carcinoma have been reported, and three cases of MDAs combined with renal cancer have been documented [2-4]. In this manuscript, we present a unique case of uterus didelphys with concurrent endometrial cancer in a patient who had previously undergone treatment for clear cell renal carcinoma eight months prior.

## Case presentation

A 54-year-old gravida 1 para 1 female patient presented for gynecological evaluation due to postmenopausal vaginal bleeding. The patient had a history of left radical nephrectomy, left salpingo-oophorectomy, and omental biopsy due to left renal and left ovarian tumors eight months before the current diagnosis. This operation was not conducted in our institution. The biopsy results indicated clear cell renal carcinoma pT3a, pNx, an endometrioid adenofibromatous tumor of borderline malignancy (ovarian tumor), while the omental biopsy showed no disease. The patient had no family history of malignancy.

Outpatient transvaginal ultrasound (TVUS) revealed an endometrial thickness of 7 mm with no visible endometrial lesion in the uterus, while no other anomalies were described in the report. The decision was made for the patient to undergo hysteroscopy combined with dilation and curettage (D/C). During hysteroscopy, an incidental finding of two cervixes and two endometrial cavities with a single vaginal canal was encountered. D/C was performed on both uterine cavities, and endometrial sampling was taken for further analysis. Pathology results confirmed the lesion as endometrioid adenocarcinoma grade 1.

**Figure 1:**
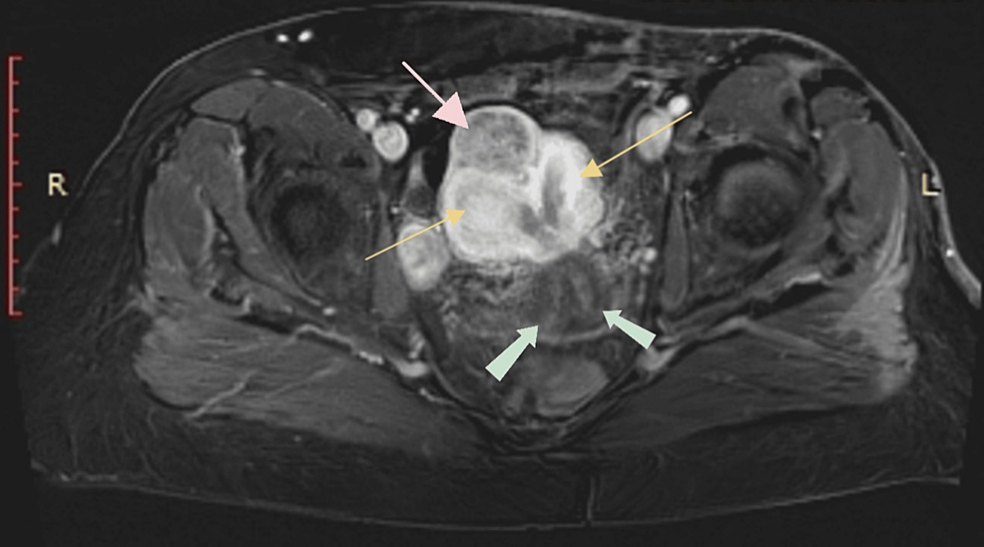
MRI T1-weighted examination showing uterus didelphys (yellow arrows), two cervixes (green arrows), and a uterine leiomyoma (pink arrow - axial plane).

After the D/C, outpatient preoperative MRI staging showed a double cervix with a common upper cervical canal and two separate endometrial cavities, which united at the inferior common endometrial canal (Figure 1). Moderate enhancement was detected in the common endometrial cavity, the entire right endometrial cavity, and partially in the left cavity. No myometrial invasion was noted, and there was no evidence of pelvic, para-aortic, or inguinal lymphadenopathy. The multidisciplinary team’s decision was surgical treatment.

**Figure 2:**
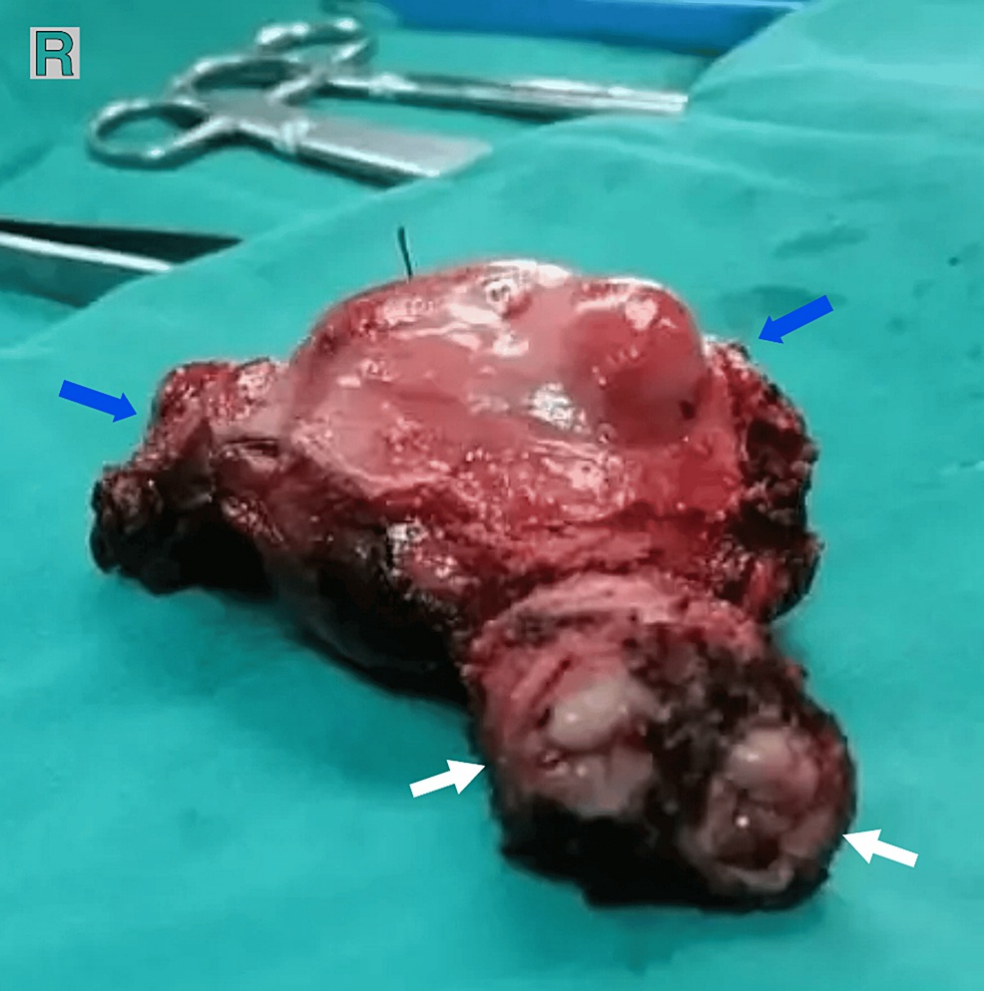
Gross specimen: double uterus 9 × 5 × 4 cm (left horn 40 mm; right horn 35 mm) (blue arrows) with two cervixes (white arrows).

**Figure 3:**
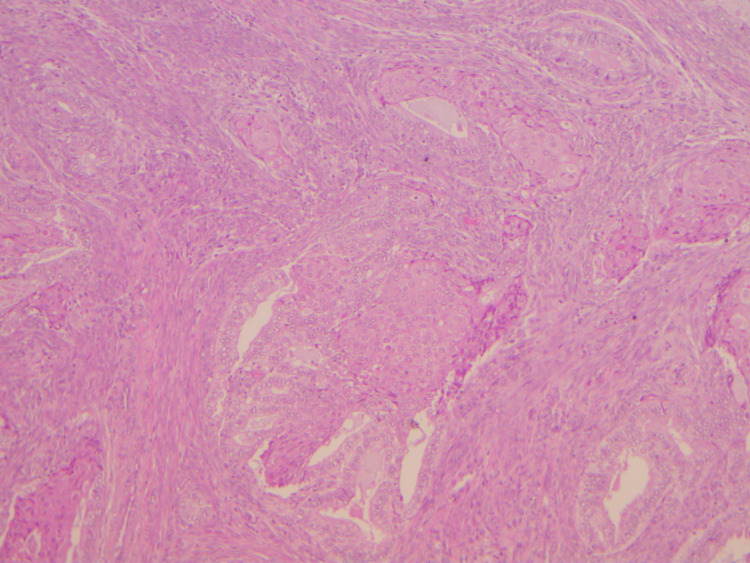
Endometrioid adenocarcinoma (grade 1), hematoxylin and eosin staining (×100) showing squamous metaplasia within the myometrium.

For the surgery, a midline incision was chosen, and the patient underwent an open abdominal hysterectomy, a right salpingo-oophorectomy, and an omental biopsy, with no evidence of tumor spillage. During surgery, a didelphys-shaped uterus with two cervixes was identified. Macroscopic examination revealed a double uterus measuring 9 × 5 × 4 cm (left horn 40 mm; right horn 35 mm) (Figure 2), with two cervixes sharing one cervical canal. The pathology report confirmed the presence of a mass measuring 35 mm in diameter, which occupied the anterior right horn, anterior left horn, and inferior common endometrial cavity. The myometrial invasion was found to be less than 50%, and the diagnosis of endometrioid adenocarcinoma grade 1 was confirmed (International Federation of Gynaecology and Obstetrics (FIGO) 2018, stage IA, Figure 3). No MMR testing was performed on the specimen, as this was not the standard of care in our institution at the time of this operation.

The patient had a short and complication-free postoperative hospital stay until the day of discharge. The multidisciplinary team determined that the patient should undergo follow-up examinations, and if no remaining disease or recurrence is found, no further actions are required. As of the present date, 10 months later, the patient remains disease-free.

## Discussion

Congenital uterine anomalies are often associated with renal abnormalities, as both the genital and urinary tracts originate from the urogenital ridges, with their development closely intertwined [5]. The precise development of Müllerian ducts is believed to be influenced by the mesonephric ducts [5]. In individuals with MDAs, urinary tract abnormalities may include ipsilateral renal agenesis, duplex collecting systems, renal duplication, and horseshoe-shaped kidneys. Uterine didelphys is particularly strongly correlated with urinary anomalies, most commonly unilateral renal agenesis [1].

Our patient had a history of clear cell renal carcinoma. After an extensive literature review, we identified only three case reports of MDAs combined with renal cancer, which are presented in Table 1, alongside our case.

**Table 1:** Cases of MDAs combined with renal malignancy. BSO = bilateral salpingo-oophorectomy; G1 = grade 1; MRKH = Mayer-Rokitansky-Küster-Hauser; MDAs = Müllerian duct anomalies; N/A = not acquired; TAH = total abdominal hysterectomy

References	Age	Deformity	Malignancy	Treatment	Follow-up
Bairaktaris et al. (2023)	54	Didelphys uterus	Endometrioid (G1) uterine cancer, clear cell renal cancer	First surgery: left radical nephrectomy, left adnexectomy. Second surgery: abdominal hysterectomy, right adnexectomy	9 months free of disease
Mermerkaya et al. (2013) [2]	16	Unilateral renal agenesis, herniating ovary, MRKH syndrome	Renal cell carcinoma	N/A	N/A
Soni et al. (2012) [3]	47	Congenital absence of uterus and ovaries, partial vaginal agenesis, bilateral ectopic kidneys, MRKH syndrome (type II)	Moderately differentiated squamous cell renal carcinoma	N/A	N/A
Woods et al. (1992) [4]	61	Septate vagina, double cervix, uterus didelphys, and a single kidney	Uterine cancer, renal cell carcinoma	Pelvic irradiation, TAH, BSO, heminephrectomy	18 years free of disease

Concerning MDAs, many women with this condition remain asymptomatic and undiagnosed. Common symptoms include dysmenorrhea or dyspareunia, while rarer cases may involve genital neoplasms and hematocolpos [1]. While two-dimensional TVUS is commonly used as an initial approach, its diagnostic specificity is limited due to challenges in distinguishing between MDA subtypes. Three-dimensional (3D) TVUS is being increasingly utilized as it offers valuable information about fundus and endometrial cavity morphology through the coronal view [6]. In cases where non-diagnostic 3D TVUS results are obtained, MRI may be employed, and invasive methods such as hysteroscopy, hysterosalpingography, and laparoscopy may be necessary to diagnose urinary tract deformities [6].

In our case, the patient presented to our institution for an evaluation of postmenopausal vaginal bleeding, without any of the previously mentioned complaints. What made this routine evaluation surprising was that the outpatient TVUS report did not mention any structural uterine anomaly. Consequently, the discovery of the two cervical oses was entirely incidental during hysteroscopy. However, to maintain the true sequence of events, we have chosen to present it as is.

As previously mentioned, the patient had also sought evaluation and treatment at a different institution for renal cancer along with an ovarian tumor before presenting to us. A midline incision was chosen by the clinicians so that both renal and ovarian tumors could be approached. Apart from this operation, the patient received no further treatment. Unfortunately, no further histological or immunohistochemical analyses were conducted on these specimens, nor were they any longer available to us at the time the patient presented to our institution. Based on our assessment, we cannot establish a direct correlation between the borderline malignancy of the ovarian adenofibromatous tumor and the subsequent diagnosis of endometrial cancer.

In Table 2 below, we present a narrative review exploring the correlation between didelphys uterus and endometrial carcinoma. A comprehensive search was conducted on Medline and Scopus databases using relevant keywords such as “Didelphys Uterus,” “Double Uterus,” “Endometrial Cancer,” “Müllerian Duct Anomalies and Renal Anomalies,” and “Müllerian Duct Anomalies and Renal Cancer.” Our search identified a total of 32 reported cases of didelphys uterus combined with endometrial cancer, including our own case. These cases were published between 1957 and 2022, involving patients aged between 30 and 78 years (Table 2).

**Table 2:** Cases of didelphys uterus and endometrial cancer. AC = adenocarcinoma; BSO = bilateral salpingo-oophorectomy; CS = carcinosarcoma; EAC = endometrial adenocarcinoma; EC = endometrial cancer; EVS = excision of the vaginal septum; FIGO = International Federation of Gynecology and Obstetrics; G1 = grade 1; G2 = grade 2; G3 = grade 3; IOB = infracolic omental biopsy; LSO = left salpingo-oophorectomy; MRH = modified radical hysterectomy; N/A = not available; OMT = omentectomy; RSO = right salpingo-oophorectomy; SAC = serous adenocarcinoma; SN = sentinel node; SC = sarcoma; SH = simple hysterectomy; TAH = total abdominal hysterectomy

References	Age	Surgery	Pathology	Staging
Bairaktaris et al. (2023)	54	TAH, RSO, Omental biopsy	Endometrioid (G1) EC	IA FIGO 2018
Chen et al. (2022) [7]	40	Laparoscopic MRH, pelvic lymphadenectomy, and abdominal para-aortic lymphadenectomy	Endometrioid (G1) EC	IB FIGO 2018
Yu et al. (2021) [7]	30	Left uterus and horn resection	Endometrioid (G2) EC	IA FIGO 2018
Kobayasi et al. (2021) [7]	58	Robot-assisted hysterectomy, BSO, and pelvic lymphadenectomy	EC	N/A
Sassine et al. (2021) [8]	70	TAH + BSO + OMT, bilateral obturator SN	Endometrioid (G1) EC	IB FIGO 2018
Vanichtantikul et al. (2020) [7]	50	Laparoscopic hysterectomy	Endometrioid (G1) EC	IA FIGO 2018
Gao et al. (2017) [9]	75	TAH + BSO	CS	II FIGO 2009
Vasquez et al. (2014) [9]	59	Laparoscopic hysterectomy, BSO, pelvic and para-aortic lymphadenectomy	Endometrioid EC	IB
Iavazzo et al. (2013) [9]	73	TAH + BSO, vaginal excision of the protruding tumor	CS (G3)	IIIB
Morris et al. (2012) [10]	78	TAH + BSO	Endometrioid (G1) EC	IB FIGO 2008
Kunos et al. (2011) [9]	48	LSO	Mucinous (G1) EC	IIIA
Suprasent et al. (2010) [9]	72	SH + BSO	CS (G3) EAC + SAC + SC	II
Chen et al. (2008) [9]	41	Staging surgery	EAC (G2)	IA
Fanfani et al. (2006) [9]	33	TAH + BSO + IOB	EAC (G2)	IA
Bhalla et al. (2005) [9]	47	TAH + BSO	EAC (G2)	II
Molpus et al. (2004) [9]	59	TAH + BSO + EVS	EAC (G1)	IA
Molpus et al. (2004) [9]	66	Staging surgery	EAC (G3) with clear cell features	IIIC
Kondi–Pafiti et al. (2003) [9]	58	TAH + BSO	Endometrioid (G1) EC	IB
Holub et al. (1998) [9]	45	Staging surgery	EAC (G3)	IA
Pojman et al. (1995) [11]	78	TAH + BSO	EAC (G2)	IB
Kosinski et al. (1994) [9]	40	TAH	EAC (G1)	IB
Woods et al. (1992) [4]	63	TAH + BSO	EC + Renal Cancer	IA or IB
Vial’tsev et al. (1991) [12]	52	N/A	Endometrioid (G1) EC	N/A
Vial’tsev et al. (1991) [12]	48	N/A	EC (G1)	N/A
Tsukuhara et al. (1990) [11]	61	TAH + BSO	Papillotubular EAC (G1)	IVB FIGO 2018
Thomas et al. (1988) [13]	N/A	N/A	EC	N/A
Eichner et al. (1981) [14]	N/A	N/A	EAC	N/A
Anneberg (1971) [15]	N/A	N/A	EAC	N/A
Braun et al. (1970) [14]	N/A	N/A	EC	N/A
Assen (1970) [16]	N/A	N/A	EC	N/A
Grant et al. (1970) [17]	N/A	N/A	EAC	N/A
Fealy et al. (1957) [18]	N/A	N/A	EAC	N/A

Notable highlights from the presented table include two unique cases, namely, fertility-sparing treatment and sentinel lymph node (SLN) mapping. Up to this point, fertility preservation in cases of endometrial cancer and didelphys uterus has been reported in just one documented case. This case involved a 30-year-old female diagnosed with endometrioid carcinoma in the left horn of a didelphys uterus. After initial conservative treatment with megestrol acetate proved unsuccessful, she underwent abdominal surgery. During the surgery, the left uterus and fallopian tube were resected (staged IA, grade 2), while the healthy horn was preserved. The patient experienced a smooth recovery, with no reported abnormalities during the close follow-up period, during which she attempted to conceive through a fertility center [7].

Additionally, the utilization of SLN mapping in cases of didelphys uterus combined with endometrial cancer has been documented in a single case report by Sassine et al. [8]. In this instance, the patient, a 70-year-old female diagnosed with endometrioid adenocarcinoma in the left uterine horn (stage IB, grade 1, FIGO 2018), underwent SLN mapping. The procedure involved an injection of indocyanine green at specific cervical sites, following National Comprehensive Cancer Network guidelines. Subsequently, the patient underwent a total abdominal hysterectomy, bilateral salpingo-oophorectomy, omentectomy, pelvic washings, and the removal of both obturator SLNs (with negative biopsy results). According to the latest follow-up, the patient remains free of disease six months after the procedure.

However, due to the small number of published cases so far, no established correlation between MDAs and endometrial or renal cancer could be made. Future analysis with more available data could show whether there is a risk for such concurrent malignancies in women diagnosed with MDAs.

## Conclusions

Our case report highlights the rare co-occurrence of endometrial cancer in both horns of a didelphys uterus in a patient with a history of renal cancer. The incidental discovery of the didelphys uterus during the evaluation for postmenopausal vaginal bleeding underscores the importance of thorough diagnostic investigations, even in routine clinical scenarios. The association between MDAs, such as didelphys uterus, and the development of endometrial and renal cancer warrants further research to elucidate potential correlations. While our manuscript adds to the limited literature on this subject, it also emphasizes the need for heightened awareness among healthcare providers, radiologists, and gynecologists to consider the possibility of MDAs in their diagnostic assessments, even when not initially indicated. Continued exploration of such cases may contribute to a better understanding of the complex interplay between MDAs and cancer, ultimately benefiting patient care and management.

## References

[1] Wu CQ, Childress KJ, Traore EJ, Smith EA (2021). A review of Mullerian anomalies and their urologic associations. Urology.

[2] Mermerkaya M, Burgu B, Hamidi N (2013). Mayer-Rokitansky-Küster-Hauser syndrome accompanied by renal cell carcinoma: a case report. J Pediatr Hematol Oncol.

[3] Soni HC, Jadav VJ, Sumariya B, Venkateshwaran KN, Patel N, Arya A (2012). Primary malignancy in crossed fused ectopic kidney. Abdom Imaging.

[4] Woods MS, Sheppard RG, Hardman DA, Woods HJ (1992). Congenital genitourinary anomalies. Is there a predilection for multiple primary malignant neoplasms?. Cancer.

[5] Mooren ER, Cleypool CG, de Kort LM, Goverde AJ, Dik P (2021). A retrospective analysis of female Müllerian duct anomalies in association with congenital renal abnormalities. J Pediatr Adolesc Gynecol.

[6] Takagi H, Matsunami K, Noda K, Furui T, Imai A (2003). Magnetic resonance imaging in the evaluating of double uterus and associated urinary tract anomalies: a report of five cases. J Obstet Gynaecol.

[7] Chen L, Zhang F, Ma YB, Chen JL (2022). Uterus didelphys complicated with endometrial carcinoma: a case report of uterus didelphys with endometrial carcinoma. Medicine (Baltimore).

[8] Sassine D, Moufarrij S, Hodgson A, Ehmann S, Abu-Rustum NR, Chiang S, Jewell EL (2021). Case report: sentinel lymph node mapping of endometrial carcinoma occurring in uterine didelphys. Gynecol Oncol Rep.

[9] Gao J, Zhang J, Tian W (2017). Endometrial cancer with congenital uterine anomalies: 3 case reports and a literature review. Cancer Biol Ther.

[10] Morris L, Stevens MJ, Valmadre S, Martland J, Lee T (2012). Adjuvant intravaginal brachytherapy for uterus didelphys with synchronous endometrial adenocarcinomas and unfavourable vaginal topography. Gynecol Oncol Case Rep.

[11] Pojman DV, Taxy JB (1995). Images in clinical medicine. Double uterus with adenocarcinoma. N Engl J Med.

[12] Vial'tsev NV, Sokolova NV, Pronin AG (1991). [Development of endometrial cancer in one half of a double uterus]. Arkh Patol.

[13] Thomas AG, Deligdisch L, Goldstein M (1988). Endometrial carcinoma with uterus didelphys. Mt Sinai J Med.

[14] Eichner E, Simak KA (1981). Uterus didelphys unicollis with adenocarcinoma in one horn and atypical endometrial hyperplasia in the other: case report. Am J Obstet Gynecol.

[15] Anneberg AD (1971). Double vagina with double uterus (didelphys) containing endometrial adenocarcinoma. Report of a case. J Iowa Med Soc.

[16] van Assen FJ (1970). An exceptional case of endometrial carcinoma or a double tumour of the uterus?. Ned Tijdschr Verloskd Gynaecol.

[17] Grant KB, Sedlacek RL (1970). Uterus didelphys with adenocarcinoma in one fundus--a case report. J Iowa Med Soc.

[18] Fealy J, Nelson JH (1957). Adenocarcinoma in one-half of a uterus didelphys. Med Ann Dist Columbia.

